# Appraisal and recommendation synthesis of guidelines and consensuses for interventions of pressure ulcers: A protocol for systematic review

**DOI:** 10.1097/MD.0000000000020417

**Published:** 2020-05-22

**Authors:** Jie Geng, Ke-Lu Yang, Yi-Tong Cai, Ji-Yuan Shi, Min Yin, Xiao-Ping Wang

**Affiliations:** a Evidence-Based Nursing Center, School of Nursing, Lanzhou University; b The Second Hospital of Lanzhou University; c The First Hospital of Lanzhou University, Lanzhou; d The First People's Hospital of Baiyin, Baiyin, China.

**Keywords:** clinical practice guidelines, pressure ulcers, quality assessment

## Abstract

**Background:**

Pressure ulcers (PU) bring a considerable physical and mental burden on patients and their families, and have put families and government under tremendous pressure to cover the cost for treatment. Therefore, this protocol proposes to evaluate the quality of existing PU clinical practice guidelines (CPGs) and compare the similarities and differences between its recommendations in order to improve the treatment efficacy and reduce the PU treatment cost.

**Methods:**

Electronic databases and specific databases of CPGs will be searched. Study selection and data collection will be performed independently by two reviewers. The Appraisal of Guidelines for Research & Evaluation II (AGREE II) Instrument and Reporting Items for Practice Guidelines in Healthcare (RIGHT) will be used to assess the methodological quality and reporting quality of included CPGs. Bubble plot will be used to describe the difference of the quality, and mind mapping will be plotted to illustrate the comparison of recommendations of a guideline when needed. R software, MindMaster and Excel will be used.

**Results:**

The results of this study will be submitted to a peer-reviewed journal for publication.

**Conclusion:**

This systematic review will provide comprehensive evidence of CPGs of PU.

**PROSPERO registration number:**

CRD42020149176.

## 1 Introduction

Pressure ulcers (PU) is localized damage to the skin and underlying soft tissue usually over a bony prominence or related to a medical or other devices.^[1,2]^ PU may cause patients considerable pain and discomfort associated with wounds, and has been considered as an outcome of poor-quality nursing care.^[3,4]^ The prevalence rate of PU varies among countries of different levels of economic development around the world, such as Ethiopia, one of the low-income countries, even reaching 14.9%, and the same region or district shows variety over the time.^[5–8]^ PU shows high incidence rate in patients undergoing surgery, and in intensive care unit (ICU) or geriatrics wards (1.9%–53.4%).^[1]^ Cost of PU prevention per patient per day varied between 2.65 € to 87.57 €, as well as treatment per patient per day ranged from 1.71 € to 470.49 € across different settings, and the annual cost has reached €2.5 billion in Europe.^[7,9,10]^ A considerable physical and mental burden has been brought on patients and their families, and families and government have been put under tremendous pressure to cover the cost for treatment. Numerous different interventions have been involved in the prevention and management of people with PU including nutritional care, pressure reducing/relieving surfaces, skin and wound care, repositioning, and risk assessment tools.^[11–14]^ Whereas many aspects of prevention and treatment, including time points, operation processes, and dressing selection, have been yet to be standardized.^[15–17]^ Hence, if the current best evidence has been timely and accurately used, it may improve the treatment efficacy and reduce the PU treatment cost.

Making use of the best evidence is a fundamental aspect of quality health care, and clinical practice guidelines (CPGs) are at the top of an evidentiary pyramid in evidence-based medicine.^[18–20]^ With the rapid increase in the number of CPGs, many countries and agencies have developed various CPGs on the same subject. Nevertheless, different academic organizations, different target population as well as settings, and the diversity of inclusion criteria and evidence rating systems have led to uneven methodological and reporting quality in CPGs, and even not exactly same recommendations on account of different emphases.^[21]^ For example, CPG published by Japanese Society of Pressure Ulcers in 2014 recommended 30- or 90-degree lateral decubitus could help reduce stress ulcers formation, but, conversely, 90-degree lateral decubitus was not recommended in CPGs published by National Pressure Ulcer Advisory Panel, European Pressure Ulcer Advisory Panel and Pan Pacific Pressure Ulcers Alliance in 2014.^[1,22]^ Reposition is an important intervention to relieve pressure and provide an adequate supply of oxygen and nutrients, so different angle of lateral decubitus position may cause different effects.^[11,16]^

Therefore, to select and apply the current best evidence to clinical work from a wide range of existing CPGs to reduce the incidence of PU and treatment costs, as well as improve the satisfaction of patients and their families are still an urgent problem to be solved. No systematic review has performed about the critical appraisal on the development of CPGs for PU. The present study will evaluate the quality of existing PU CPGs and compare the similarities and differences between its recommendations.

## 2 Methods

### 2.1 Study design

This systematic review of CPGs about diagnosis, prevention, and management of PU will be undertaken to assess the methodological quality in their development and summary the coincident recommendations, available in those documents.

### 2.2 Protocol and registration

This study will be reported according to the Preferred Reporting Items for Systematic Reviews and Meta-Analyses (PRISMA-P).^[23]^ The systematic review was registered in the International Prospective Register of Systematic Reviews (PROSPERO) database (protocol number: CRD42020149176). Ethical approval is not required because this is a literature-based study.

### 2.3 Data sources and search strategy

Search strategies will be performed on the following electronic databases: PubMed, EMBASE, Web of Science, CINAHL Complete and Cochrane Library. Specific database for clinical guidelines and consensuses will be searched, for example: The National Institute for Health and Care Excellence (NICE) (www.nice.org.uk), Scottish Intercollegiate Guidelines Network (SIGN) (https://www.sign.ac.uk/), National Pressure Ulcer Advisory Panel (NPUAP) (https://npuap.org/), Registered Nurses Association of Ontario (RNAO) (https://communities.rnao.ca/), European Pressure Ulcer Advisory Panel (EPUAP) (http://www.epuap.org/), and so on. The MeSH search and text word search will be used with the terms related to the guideline, best practices, pressure ulcers, pressure ulcer, and decubitus ulcer. The specific search strategy will be (taking PubMed as an example) is shown in Table 1. The strategy will be modified for other databases use if necessary.

**Table 1:**
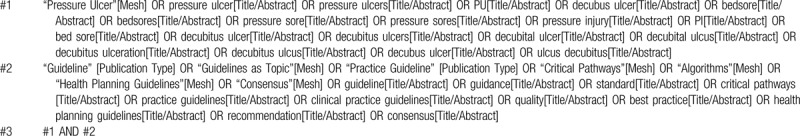
Searching strategy in PubMed.

The reference lists of eligible studies will be checked by reviewers in order to identify other possible guidelines. For guidelines published only in summary or where important information is missing, we will try to search complete information by contacting the authors.

### 2.4 Eligibility criteria

#### 2.4.1 Inclusion criteria

CPGs describing prevention, diagnosis, and management of patients of any age who already has or at risk of PU, will be included. There is no time limitation, and language is restricted to English and Chinese.

#### 2.4.2 Exclusion criteria

The available version is incomplete or contains only a summary of the information; the document is the translation of a guideline published in another language; and if there is a consensus guideline, evidence summary or algorithm; will be excluded.

### 2.5 Measured outcomes

To form a collection of consistent recommendations and inconsistent recommendations covering diagnose, prevention, treatment, and management for practicing clinicians, nurses, care-givers and patients, by comparing and synthesizing of recommendations provided by the CPGs. The methodological and reporting quality of CPGs will also be assessed.

### 2.6 Determination of eligibility

Literature search records will be imported into EndNote X8 literature management software (Thomson Reuters [Scientific] LLC, Philadelphia, PA). Two reviewers are about to screen the titles and abstracts of retrieved studies to identify potentially eligible studies. Then they will select full-text of potentially eligible studies and determine study for inclusion or exclusion. All the works above will be done independently. Any disagreement will be resolved by the third party. The selection process will be summarized according to PRISMA flow diagram.

### 2.7 Data extraction

First, a predesigned data extraction form is to be designed by our team. Then, 1 to 5 included studies will be pre-extracted. If necessary, the forms shall be continually modified until the final data extraction form complete. Two reviewers will independently extract data from each included study. Different opinions will be resolved through discussion or consult the third party.

The following items will be extracted:

(1) General characteristics: number of authors, year of publication, update time, organizations, or others;
(2) Specific characteristics: stage of PU, target population, type of studies included, methods of classifying the quality of evidence and recommendations, or others;
(3) Recommendations;
(4) Degree of recommendation;
(5) Level of evidence, or other.

### 2.8 Quality assessment of clinical practice guidelines

At least 2 reviewers will evaluate the methodological quality of the included CPGs by using the Appraisal of Guidelines for Research & Evaluation II (AGREE II) Instrument. Each of the 23 items targets various aspects of practice guideline quality. The AGREE II also includes 2 final overall assessment items that require the appraiser to make overall judgments of the practice guideline while considering how they rated the 23 items.^[24,25]^ The quality of each guideline will be calculated for each domain, according to the AGREE II User Manual. The 6 domains are independents and the scores should be calculated as the sum of the individual items in each domain. Then, the total obtained will be presented as a relation percentage to the maximum possible score for each domain.^[25,26]^

The reporting quality will be assessed by reporting items for practice guidelines in healthcare (RIGHT), which includes 22 items and seven domains.^[27]^ Any disagreement will be resolved through discussion or consultation in the third part.

### 2.9 Data synthesis

The descriptive statistics will be calculated for RIGHT as mean (standard deviation) and median (interquartile range). A quality score is calculated for each of the 6 AGREE II domains. The 6 domain scores are independent and should not be aggregated into a single quality score. Domain scores are calculated by summing up all the scores of the individual items in a domain and by scaling the total as a percentage of the maximum possible score for that domain.

Bubble plot will be used to describe the difference of the quality, and mind mapping will be plotted to illustrate the comparison of recommendations of a guideline when needed. R software, MindMaster and Excel will be used. If sufficient evidence is available, we will plan to conduct subgroup analyses to explore the difference between different editions, different institutes, and different type of guidelines.

## 3 Discussion

This study will identify CPGs of PU patients, explore the methodological quality and reporting quality of included CPGs, and assess agreement among diagnosis, prevention and management of PU to inform consistent and inconsistent recommendations. A description of the available consistent recommendations on interventions and evidence supporting them contributes to the choice of prevention and treatment for patients with PU or at risk. The inconsistent recommendations may show the future directions for research on PU. The results of methodological quality and reporting quality may show the quality status of CPGs on PU field, and indicate the areas in need of improvement.

The results of the research will be submitted for publication in scientific journals, peer reviewed, and also published in national and international conferences.

## Acknowledge

We thank the assistance of Dr. Jin-Hui Tian and Dr. Lin Han to improve the methodology and quality of language in our manuscript.

## Author contributions

JG, KLY, YTC, and XPW conceived the study, developed the criteria, JG, KLY, YTC, and MY searched the literature, and analyzed the data. JG, KLY, YTC, JYS, and XPW wrote the protocol and revised the manuscript. All authors have read and approved the final manuscript.
